# Effect of relaxation therapy on benzodiazepine use in patients with medically unexplained symptoms

**DOI:** 10.1186/s13030-020-00187-7

**Published:** 2020-07-09

**Authors:** Kazuaki Hashimoto, Takeaki Takeuchi, Akiko Koyama, Miki Hiiragi, Shunsuke Suka, Masahiro Hashizume

**Affiliations:** 1 Department of Psychosomatic Medicine, Toho University Graduate School of Medicine, 5-21-16 Omori-Nishi, Ota-ku, Tokyo, 143-8540 Japan; 2 Department of Psychosomatic Medicine, Toho University School of Medicine, Tokyo, Japan

**Keywords:** Autogenic training, Benzodiazepine dependence, Medically unexplained symptoms, Psychosomatic medicine, Psychiatry, Relaxation, Biofeedback

## Abstract

**Background:**

The change in the benzodiazepine (BZD) use of patients with medically unexplained symptoms (MUS) following the application of relaxation therapy were examined.

**Methods:**

Of the 221 outpatients with MUS using BZD, 42 received relaxation therapy. Change in BZD use was compared using a relaxation group (*n* = 42) and a control group that had 84 MUS patients whose baseline was matched by optimal matching algorithms. Logistic regression analysis was done to evaluate the effect of BZD-dependent factors on the BZD dose of the relaxation group.

**Results:**

Compared with the control group, the number of patients who decreased the amount of BZD and the number of patients whose subjective symptoms of MUS improved were significantly higher in the relaxation group (*p* < 0.05). In addition, a factor that made it difficult to reduce the BZD of MUS patients who had undergone relaxation was a long history of BZD use, for more than 6 months (odds ratio, 0.06, 95% confidence interval, 0.01–0.36).

**Conclusions:**

Relaxation therapy for patients with MUS may help reduce BZD use; however, early intervention is important to prevent BZD dependence.

## Introduction

Medically unexplained symptoms (MUS) comprise a clinical spectrum, and often cause mental and physical impairment. Cases of refractory MUS sometimes become persistent or chronic; therefore, it has been proposed that the treatment of MUS should be interdisciplinary because it has a complex clinical spectrum [1]. In our previous study, we reported various clinical symptoms of MUS [2] that complicate its treatment.

Combined use of pharmacological treatment with non-pharmacological therapies, such as psychotherapy, lifestyle changes, and relaxation therapy, are required to effectively treat MUS [3]. Autogenic training (AT) [4, 5] and biofeedback (BF) are relaxation methods with possible therapeutic effects because of their ability to strengthen patients’ self-control. Moreover, these methods have been previously reported to be effective for the treatment of various functional diseases (e.g. functional headaches) [6, 7]. Progressive muscle relaxation (PMR) [8] is another type of relaxation method in which the patients carefully classify each muscle group in the body and then repeatedly tense and relax each muscle group. PMR is a popular treatment for insomnia [9] with reported efficacy to reduce symptoms of anxiety in patients with schizophrenia, thereby improving the quality of life [10]. Usually, relaxation therapies are more effective when combined with other treatment modalities [11, 12]. For instance, in our own department, AT, BF, and PMR have been used alone or in combination as a module for cognitive-behavioral therapy. However, we previously reported that the combination of BF and PMR demonstrated superior effectiveness in the treatment of migraine [13].

Besides relaxation therapies, 19.4% of MUS cases are treated pharmacologically [14]. Benzodiazepines (BZD) are used the alleviation of the emotional component of pain because of their anxiolytic and hypnotic actions and for palliative treatment of symptoms in cases of functional diseases (e.g. tension headaches) [15] and chronic pain [16]. In most cases, BZD is prescribed after the onset of physical symptoms [17]. However, prolonged use of BZD can result in biological dependence [18, 19]. Recently, evidence from animal studies suggest that reduced expression of metabotropic glutamate receptors in the cerebral cortex may be related to BZD dependence [20]. In addition, various factors have been associated with BZD dependence. Such as use of a high dose, history of long-term use, combined use with antidepressants and short-acting BZD [21, 22].

The prescription of BZD is dictated by dosage guidelines [23]; however, adherence to these guidelines is often clinically difficult. For example, although short-term use of BZD is recommended, a previous epidemiological survey [24] reported that about 2% of adults used BZD for a year or more. A recent study from Germany [25] also indicated that an estimated 1.6 million individuals have BZD dependence, approximately equivalent to 2% of the German population.

Relaxation therapy has been reported to reduce BZD use, thus reducing the risk of BZD dependence [26]. However, no reports have assessed the association of relaxation therapy and BZD use in patients with MUS. In the present study, we evaluated the change in BZD use of patients with relaxation therapy and assessed the mechanism of these changes by examining BZD-dependent factors associated with MUS.

## Methods

### Study design

In the present retrospective cohort study, the medical records of all patients with MUS who received continuous treatment and used BZD at the Department of Psychosomatic Medicine, Toho University Omori Medical Center, between May 2010 and September 2018, were examined. MUS is defined as “physical symptoms for which no clear or consistent organic pathology can be demonstrated” [1]. Our patients were diagnosed by physicians specializing in psychosomatic medicine, as described previously [2]. Patients with unknown BZD use history (eg, those prescribed at another hospital) were excluded. The data of subjects who had relaxation introduced to their treatment was extracted for inclusion in a relaxation groups. To evaluate the effect of relaxation therapy, subjects matching the baseline of the relaxation group were selected as a control group. Control patients with MUS who were matched for age, sex, marital status, education history, drinking and smoking history, usage of antidepressants, usage of short-acting BZD and long-term use history of BZD were selected using optimal matching algorithms in a 1:2 ratio. The comparison of the the groups evaluated the number of people with improved clinical symptoms of MUS and those with reduced or increased BZD usage during a clinical course of about 10 weeks.

Furthermore, in the relaxation group, logistic regression analysis was used to evaluate the association between four factors previously associated with BZD dependence [21, 22] (dose of BZD used, history of long-term use, antidepressants, and short-acting drugs) and reduced BZD use.

All study procedures were conducted with the approval of the Toho University Medical Center Omori Hospital Ethics Committee (approval number: M18248, M19248 18249), with due consideration of the Helsinki Declaration, patient anonymity, and ethics.

### Benzodiazepine

To calculate the dose of BZD used in 1 month, the dose of BZD used was converted to the dose of diazepam, as described previously [27]. A similar method [27] was used in cases in which combinations of BZD were used. For logistic regression analysis, the dose used was converted into categorical variables (every 120 mg/month) as per standard diazepam dosing in Japan. Patients with more than a 6-month history of BZD use were defined as long-term internal patients [28]. In addition, drugs with half-lives of less than 24 h were classified as short-acting drugs, which included, triazolam, zolpidem, zopiclone, eszopiclone, etizolam, clotiazepam, brotizolam, lormetazepam, and rilmazafone.

### Relaxation therapy

Relaxation therapy was provided individually, approximately once a week for about 10 weeks, combining AT, BF, and PMR methods. AT is based on passive concentration of physical perceptions and consists of tranquility (feeling of calmness). The first exercise was heaviness exercise; repetition of the statement, “my right arm is heavy; my left arm is heavy”: and the second was warmth exercise; repetition of the statement “my right arm is warm; my left arm is warm.” PMR consisted the application of continuous tension for approximately 5 seconds followed by relaxation of each muscle group with eight repetitions. Each session, including muscle tension and relaxation were performed twice, moving from the upper body to the lower body. Basic versions of AT and PMR were performed by physicians trained according to their respectively developed style [4, 5, 8]. BF training was performed in combination with AT or PMR and comprised a 30-min session of measurement and feedback of temperatures from the first finger of the dominant hand or the measurement of mitral muscle tension by polygraph. (NeXus-4; MindMedia BV., Herten, The Netherlands). Subjects were provided with electromyogram or visual temperature feedback. The use of same Japanese relaxation therapy training protocols ensured consistency between doctors.

### Data analysis

The Fisher’s exact test was used for the categorical variables, and the Mann-Whitney U test and Student’s t-test was used for the continuous variables. Furthermore, we performed logistic regression analysis with increased or decreased BZD use as an objective variable and the dose of BZD used, history of long-term use, use of antidepressant and short-acting drug as the explanatory variables. All statistical analyses including the matching process were performed with the EZR Ver 1.32 statistical package [29]. Two-tailed *P*-values of less than 0.05 were considered statistically significant.

## Results

Of 223 MUS patients using BZD who received continuous treatment during the study period, 44 received relaxation therapy. Two patients who dropped out of our relaxation sessions were excluded, leaving the data of 42 patients, extracted from the medical record, available for the analysis of the relaxation group. Twenty eight of these patients (66.7%) were referred by their previous doctor for alternative treatment. The average age was 47.8 years. A total of 17 patients (40.4%) had an advanced degree (from university or graduate school) and 21 (50.0%) had been married. Eight patients (19.0%) had a habit of drinking and two (4.7%) had a habit of smoking. Seventeen patients (40.4%) had used antidepressants for over 1 month. The average BZD usage rate before introducing relaxation sessions was 174.0 mg/month (equivalent DAP dose), and 26 patients (61.9%) had a history of long-term use. A total of 24 used short-acting type BZD (57.1%). Eventually, 40 (95.2%) were treated with AT, 33 (78.5%) with BF, and 26 (61.9%) with PMR. Multiple relaxation therapy modalities were used in 36 cases (85.7%). Patients underwent an average of 9.5 relaxation sessions.

**Fig. 1:**
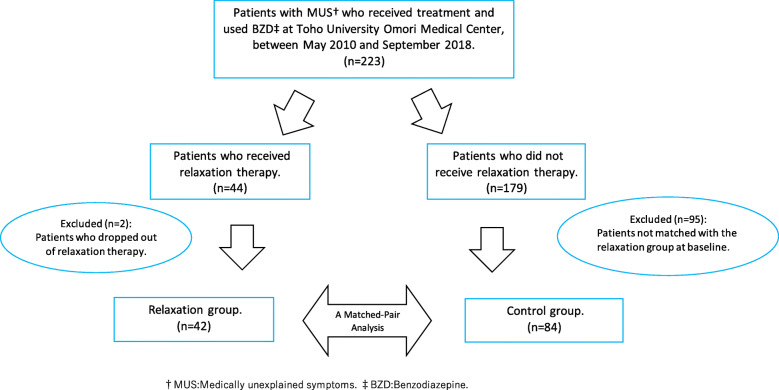
Flow chart of the study participation

A total of 42 patients included in the relaxation group were baseline-matched with 84 control patients, taken from 179 MUS patients who had been prescribed BZD but who did not receive relaxation therapy during the same period (Fig. 1).

At baseline before matching, the relaxation group had significantly fewer smokers (*p* < 0.05) and fewer antidepressant users (*p* = 0.06) than the control group. After matching, there was no difference in baseline between the two groups (Table 1).

**Table 1:** Patient characteristics

	Baseline			Matched-pair baseline		
	Control (*n* = 179)	Relaxation (*n* = 42)	*P* value	Control (*n* = 84)	Relaxation (*n* = 42)	*P* value
Sex			0.38			1.00
Male	75	14		28	14	
Female	104	28		56	28	
Age (years)	47.73 ± 15.89	47.80 ± 17.29	0.95	46.44 ± 17.29	47.80 ± 17.29	0.66
Habit						
Smoking	40	2	< 0.01	4	2	1.00
Drinking	35	8	1.00	16	8	1.00
Education (over 16 years)	65	17	0.72	31	17	0.70
Marriage	91	21	1.00	42	21	1.00
Antidepressant	102	17	0.06	34	17	1.00
Benzodiazepine						
DAP^a^ dose (mg/month)	150.00	143.35	0.24	150.00	143.35	0.76
Short acting	105	24	0.86	42	24	0.57
Long duration^b^	121	26	0.47	48	26	0.70

^a^DAP, diazepam. ^b^A long duration was defined as exceeding 6 months

Table 2 shows a comparison of the clinical symptoms of MUS and change in the use of BZD between the groups. The number of subjects who reported an improvement in the subjective clinical symptoms of MUS was significantly higher in the relaxation group (*p* < 0.01). In addition, the number of subjects who decreased BZD use was significantly higher in the relaxation group than in the control group (*p* < 0.01). On the other hand, the number of people who increased BZD use during the same period was higher in the control group (*p* = 0.08).

**Table 2:** Comparison of patient numbers for changes in clinical course (*n* = 126)

	Matched-pair		
	Control (*n* = 84)	Relaxation (*n* = 42)	*P* value
Benzodiazepine usage			
decreased (%)	8 (9.5)	13 (31.0)	< 0.01
increased (%)	14 (16.7)	2 (4.8)	0.08
Improved subjective symptoms with MUS^a^(%)	12 (14.3)	20 (47.6)	< 0.01

^a^MUS, medically unexplained symptoms

The results of logistic regression analysis examining the influence of BZD-dependent factors on decreased BZD use after relaxation therapy are shown in Table 3. Among the BZD-dependent factors, a history of long-term BZD use negatively predicted decreased BZD consumption after relaxation therapy (odds ratio [OR], 0.06, 95% confidence interval [CI], 0.01–0.36). An adjusted regression analysis yielded a similar result (OR, 0.04; 95% CI, 0.01–0.37).

**Table 3:** Factors related to decreased benzodiazepines use (*n* = 42)

Independent Variables	Comparison	Crude analysis		Adjusted analysis^a^		Adjusted analysis^b^	
		OR (95% CI)	*P* value	OR (95% CI)	*P* value	OR (95% CI)	*P* value
Dose used	DAP ^d^120 mg/month increase	0.48 (0.13–1.79)	0.27	0.45 (0.11–1.78)	0.25	0.25 (0.04–1.62)	0.15
Duration since use	≦6 months vs 6 months<	0.06 (0.01–0.36)	< 0.01	0.04 (0.01–0.29)	< 0.01	0.04 (0.01–0.37)	< 0.01
Concomitant use of antidepressants	Yes vs no	1.66 (0.31–9.05)	0.56	1.26 (0.18–8.88)	0.81	2.32 (0.20–26.10)	0.49
Use of short-acting BZD^c^	Yes vs no	2.54 (0.41–15.90)	0.32	2.53 (0.38–16.60)	0.33	5.05 (0.48–53.10)	0.18

^a^Adjusted for sex and age. ^b^Adjusted for sex, age, and relaxation therapy modality. ^c^Benzodiazepine. ^d^ Diazepam. *OR* odds ratio, *CI* confidence interval

## Discussion

In the present study, we found an association between relaxation therapy and decreased BZD use in patients with MUS. AT is an effective relaxation therapy for sleep disorders, anxiety disorders, and MUS, such as tension-type headaches, migraine headaches, and irritable bowel syndrome [30, 31]. In addition, the use of these approaches has demonstrated improved quality of life in cases of multiple sclerosis [32] and reduced anxiety and stress in healthy students [33], with one meta-analysis reporting relief from anxiety [34]. Relaxation therapy may include cognitive-behavioral therapy, and its use in this study demonstrated a positive effect on BZD use, possibly because of its multifaceted therapeutic effects on the symptoms of mind and body. Furthermore, the therapeutic utility of combining multiple relaxation modalities has been reported previously [11, 12]. Thus, combining AT, BF, and PMR in the treatment of some patients in this study may have enhanced the therapeutic effect of relaxation therapy.

Moreover, in the present study, we found that long-term BZD use affected the subsequent use of BZD in patients with MUS, making it difficult to reduce its dose. Long-term BZD use has been reported to be the most significant factor [19], suggesting that relaxation therapy would be most effective when patients with MUS start it in the early stage of BZD use of BZD. Furthermore, the results reported in this study support the existing knowledge of BZD dependency; however, two participants increased BZD use after relaxation therapy. The reason for the increased use could be due to the ineffectiveness of relaxation, which may have worsened the psychological effects of the patients, like anxiety and disappointment. In addition, the BZD dose given was unchanged for some of the patients with relaxation therapy, and further trials will be necessary to verify and assess the efficacy of relaxation therapy.

Based on previous reports, antidepressants may be useful to prevent BZD dependency [35]; however, in our study, these variables showed no significant association. The patients assessed herein might already have been treatment-resistant for antidepressants, including previous treatment.

In addition, although the use of short-acting BZD is reportedly associated with a high risk of BZD dependence [22], we observed no significant association between this factor and decreased BZD use. Short-acting BZD may cause mental dependence because of its frequent dosing, thus promoting inappropriate and frequent use. However, for some participants, relaxation behaviors may replace the inappropriate frequent use behaviors and contribute to a positive therapeutic effect, thereby affecting the analysis.

The present study has several limitations that warrant discussion. First, combined relaxation therapy was not used in isolation, and therefore, our interpretation of its effects is limited. More than 95% of the medical facilities in Japan utilize a multiple relaxation modality approach [36], which presents a potential problem with the systematic standardization of medical treatment. To compensate, we performed the analysis after adjusting for the different relaxation modalities. Furthermore, the small number of high-dose BZD users in the present study may be a limitation of the clinical sample used herein. Moreover, this study was performed at a single institution and included only a control group without relaxation therapy; therefore, it may not be possible to determine if relaxation therapy was more effective than other treatments for reducing BZD use. In the future, it will be beneficial to carry out studies by performing sample homogenization with a multi-institutional research approach and comparison of the cost effectiveness of relaxation therapy with other behavioral therapies or medication for treating MUS.

## Conclusions

We examined the effect of relaxation therapy for MUS on the BZD use in a cohort of Japanese patients. We found decreased BZD consumption with relaxation therapy. Furthermore, a history of long-term BZD use was negatively related to BZD dose reduction. To reduce the dose of BZD and to avoid BZD dependency by patients with MUS, relaxation therapy should be introduced early in the course of symptom progression.

## Data Availability

We are not able to share the current study data because sharing data is not permitted by our hospital ethics committee.
